# Health Education Through a Campaign and mHealth to Enhance Knowledge and Quality of Life Among Patients With Chronic Kidney Disease in Bangladesh: Protocol for a Randomized Controlled Trial

**DOI:** 10.2196/30191

**Published:** 2021-11-19

**Authors:** Mohammad Habibur Rahman Sarker, Michiko Moriyama, Harun Ur Rashid, Md Moshiur Rahman, Mohammod Jobayer Chisti, Sumon Kumar Das, Yasmin Jahan, Samir Kumar Saha, Shams El Arifeen, Tahmeed Ahmed, A S G Faruque

**Affiliations:** 1 Graduate School of Biomedical and Health Sciences Hiroshima University Hiroshima Japan; 2 Kidney Foundation Hospital and Research Institute Dhaka Bangladesh; 3 Nutrition and Clinical Services Division icddr,b Dhaka Bangladesh; 4 Menzies - School of Health Research Charles Darwin University Darwin Australia; 5 Child Health Research Foundation Dhaka Shishu Hospital Dhaka Bangladesh; 6 Maternal and Child Health Division icddr,b Dhaka Bangladesh

**Keywords:** chronic kidney disease, campaign, mHealth, knowledge, Bangladesh

## Abstract

**Background:**

Despite the growing burden of chronic kidney disease (CKD), disease knowledge and understanding are still lacking, especially in Bangladesh.

**Objective:**

The aim of this study was to evaluate the outcome of a health education intervention in order to enhance knowledge, health-related quality of life (QOL), and motivation regarding healthy lifestyles among rural and periurban adults suffering from CKD.

**Methods:**

A parallel-group (1:1) randomized controlled trial is ongoing in the Mirzapur subdistrict, Bangladesh, where two groups of patients with CKD are being compared. Patients aged 18 years and over with CKD (stages 1-3) were enrolled in November 2020. Patients were randomly allocated into either the intervention group (n=63) or the control group (n=63). The control group received usual treatment, while the intervention group received health education through a CKD campaign facilitated by a nephrologist and via mHealth (ie, periodic mobile phone calls) from community health workers. Both groups were followed up for a period of 6 months. The primary endpoint is patients’ increased knowledge measured using the Chronic Kidney Disease Knowledge Questionnaire. The secondary endpoints are improved QOL measured using the standardized EuroQol 5-Dimension 5-Level (EQ-5D-5L) questionnaire as well as improvements in the levels of blood pressure, BMI, serum creatinine, fasting blood sugar, hemoglobin, cholesterol, high-density lipoprotein cholesterol, triglyceride, serum uric acid, blood urea nitrogen, and albumin to creatinine ratio.

**Results:**

Enrollment of participants began in November 2020; the intervention and follow-up were completed in May 2021. We enrolled 126 patients in the study. Patients’ mean ages were 57.97 (SD 15.03) years in the control group and 57.32 (SD 14.37) years in the intervention group. There were 45 out of 63 (71%) females in the control group and 38 out of 63 (60%) females in the intervention group. In addition, there were 38 out of 63 (60%) literate patients in the control group and 33 out of 63 (52%) literate patients in the intervention group.

**Conclusions:**

It is expected that a combined approach, incorporating both a CKD campaign and mHealth, for health education may be an effective tool for increasing knowledge and improving QOL among patients with CKD.

**Trial Registration:**

ClinicalTrials.gov NCT04094831; https://clinicaltrials.gov/ct2/show/NCT04094831

**International Registered Report Identifier (IRRID):**

DERR1-10.2196/30191

## Introduction

### Background

Chronic kidney disease (CKD) is a public health concern worldwide [1] and directly affects the global burden of morbidity and mortality [2]. CKD is associated with substantial financial costs for both patients and health care systems, and low- and middle-income countries (LMIC) have a much higher economic burden of CKD than developed countries [3]. If the stage of CKD advances in patients, the cost of medical treatment increases [4]. In LMIC, most people with kidney failure have insufficient access to lifesaving renal replacement therapy [5]. Diabetes, hypertension, obesity, and aging are the leading associated factors for CKD throughout the world [6]. These comorbidities make patients with CKD more vulnerable to end-stage renal disease (ESRD) [7].

Knowledge and self-management behaviors must be learned and practiced in order to slow the progression of CKD [8]. Studies have documented that knowledge is essential for slowing kidney function advancement through behavior adjustments, such as physical exercise, dietary changes, patient monitoring (eg, blood pressure [BP] and blood glucose), and adherence to medication [9]. Patients with CKD may benefit from knowledge to change their behavior, improve their outcomes, and lower their mortality rates [10]. Considering the increasing prevalence of CKD, only 6% of the general population and 10% of those at high risk are aware of their CKD status in LMIC [11]. This low health literacy and unawareness in the early stage of the disease are ultimately responsible for CKD disease progression [12]. It has been noted, however, that proper CKD knowledge and management as well as mitigation of CKD-related risks may delay the progression to ESRD and other interrelated health consequences [10,13]. However, most of the patient education research regarding CKD has focused on patients with ESRD. A few studies on CKD have been conducted in Bangladesh, where most were prevalence studies based on a hospitalized urban population; however, there has been no such study in rural Bangladesh describing the knowledge of CKD and health-related quality of life (QOL).

In the field of nephrology, both developed and developing countries are still implementing mobile health (mHealth) [14,15]. However, mobile phone call–based health education has great potential to provide CKD knowledge and improve QOL because it relies on basic mobile technology and requires limited literacy or numeracy skills [16]. In countries with minimal or no national health insurance, such as Bangladesh, CKD education in the early stages of the disease could be an integral part of patient management and the reduction of its related risk factors to slow down its progression; the need is greater in rural and periurban areas. Community health workers (CHWs) have the potential to make a significant difference in the community’s well-being as well as in the lives of people living with kidney disease [17]. A nephrologist-facilitated health campaign, on the other hand, has high potential for delivering CKD education. Thus, this study aimed to evaluate the outcome of a health education intervention in order to enhance knowledge, health-related QOL, and motivation about healthy lifestyle among rural and periurban adults suffering from CKD (stages 1-3).

### Hypothesis

We hypothesize that the knowledge and health-related QOL of patients with CKD will be improved by health education provided by a CKD campaign and a mobile phone call–based intervention.

## Methods

### Design

**Figure 1:**
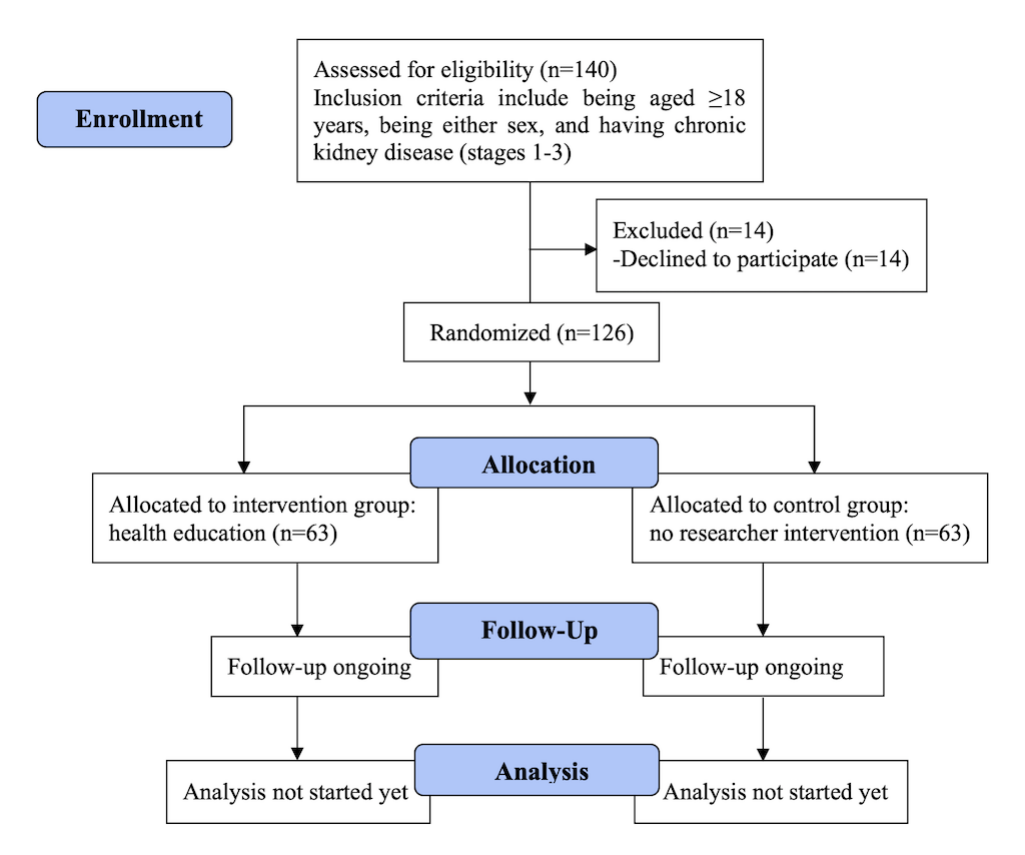
Flowchart of the study.

This study is a community-based, single-centered, prospective, open-label, two-arm (1:1), randomized control trial involving patients with CKD and is being conducted in rural and periurban areas of Bangladesh. This study has been designed in accordance with the CONSORT (Consolidated Standards of Reporting Trials) [18] and SPIRIT (Standard Protocol Items: Recommendations for Interventional Trials) guidelines [19]. The study flowchart is shown in Figure 1. The total study duration was planned to be 1 year; however, the intervention started in mid-November 2020 and the follow-up period was 6 months long. The last follow-up was completed in May 2021.

### Study Site

This study was conducted in the demographic surveillance system (DSS) area of the Mirzapur subdistrict within the Tangail district, which is located about 60 km north of Dhaka, the capital city of Bangladesh. It has a mixed rural and periurban population [20]. The subdistrict has 13 unions—it is the smallest administrative unit—and 219 villages. Among the 10 unions in Mirzapur that comprise the DSS area, three unions—Mirzapur Sadar, Bhatgram, and Gorai—nearest to our sentinel health facility, which is popularly known as Kumudini Hospital, were selected purposively due to budgetary constraints and time limitations. The study participants were required to visit Kumudini Hospital Laboratory for their study-related investigations and had lower travel costs compared to other areas.

### Study Population and Eligibility Criteria

We recruited patients for this study from among the residents of the DSS. Inclusion criteria for enrollment in the study were as follows: aged 18 years and older, either sex, diagnosed with CKD (stages 1-3: estimated glomerular filtration rate=30-59 mL/min/1.73 m^2^ and/or albumin to creatinine ratio [ACR]≥30 mg/g for more than 3 months) [21], had lived steadily in the locality for at least 5 years, had a personal cell phone or access to a shared phone, and had given written informed consent to participate. The following were excluded from the study: individuals who were hospitalized at the time of enrollment; had any known serious illness with questionable prognosis, for example, stage 4 or 5 CKD, malignancy, mental illness, congenital disease, and physical disability, if they had prescriptions; and did not agree to give consent.

### Randomization

A permuted block-randomization technique was performed using a block size of 6 based on a computer-generated random number sequence. An experienced statistician, not involved in the study in any way, prepared the randomization table and placed the study IDs of the patients with CKD, along with their corresponding intervention allocations, into serially numbered sealed envelopes according to the randomization schedule; these corresponded to the serial numbers of the patients with CKD. These envelopes were kept in an office locker. Allocations to intervention and control groups were concealed in identical sealed envelopes that were only to be opened when the study patient was ready for enrollment under the supervision of the principal investigator. This took place after a patient with CKD was recruited to the study, after obtaining voluntary informed written consent and assignment of a study ID.

### Study Activities and Content

During home visits, the CHWs used a Portable Health Clinic (PHC) box with the essential diagnostic equipment for this research; details are described elsewhere [22,23]. At baseline, CHWs performed home visits to obtain written informed consent and to interview the study patients by administering field-tested standardized questionnaires. The questionnaires collected the following data: sociodemographic information, such as age, gender, marital status, religion, occupation, educational background, and income per month; patients’ current medical status, including medication use; patients’ medical history; and patients’ family history (3rd generation), including current and immediate-past medical status. The same information was collected at 3 months and at 6 months, in case of any changes from baseline. To evaluate the level of knowledge, awareness, and QOL on the part of the study patients, the CHWs also administered questionnaires at baseline to collect this data and administered the same questionnaire at 3 months and at 6 months.

CHWs also performed physical examinations at baseline and performed them again at 3 months and at 6 months to measure the following: BP, pulse, height, weight, waist and hip circumferences, and mid–upper arm circumference. However, during baseline measurements, the study patients were advised to visit Kumudini Hospital Laboratory for collection of blood and urine samples to estimate the condition of their kidneys and their related risk factors, such as serum creatinine, fasting blood sugar (FBS), hemoglobin, cholesterol, high-density lipoprotein cholesterol (HDL-c), triglyceride, serum uric acid, blood urea nitrogen (BUN), and ACR. The same laboratory investigations were done at the end of 6 months (Table 1).

**Table 1:** Study activities.

Schedule		Intervention group	Control group
**Baseline sessions**			
	Week 1	Interview Administer questionnaires Laboratory test (blood and urine)	Interview Administer questionnaires Laboratory test (blood and urine)
	Weeks 1 and 2	CKD^a^ health campaign (3-hour lecture and discussion by a nephrologist) Provide health education materials (leaflet, short textbook, recording notebook, and a 5-gram salt measuring spoon) during campaign	Usual care
	Week 3 to month 3	Mobile education: over the telephone once every 2 weeks (5 times) Blood pressure check: once per week	Usual care
**Intermediate sessions**			
	Week 1 of month 4	Interview Administer questionnaires	Interview Administer questionnaires
	Months 4 to 6	Mobile education: over the telephone once every 2 weeks (5 times) Blood pressure check: once per week	Usual care
**Final session**			
	End of month 6	Interview Administer questionnaires Laboratory test (blood and urine)	Interview Administer questionnaires Laboratory test (blood and urine)

^a^CKD: chronic kidney disease.

### Training of CHWs

CHWs obtained written informed consent, performed physical examinations, interviewed the study participants after administering field-tested questionnaires, and provided health education. Training on the overall study procedure for CHWs was provided by the principal investigator. Training included the following: (1) general information about the study (ie, contact information, study overview, structure, and use of technology) and (2) role-specific information (ie, position description, recruitment, informed consent, data collection, use of an electronic database for data entry, proper use of backup paper copies, and self-evaluation form). Competency was assessed by practicing and role-playing in a private office. A nephrologist trained the CHWs, who then provided mHealth education to the study patients.

### Intervention Group

The intervention group received health education through a CKD campaign using mHealth technology.

#### CKD Campaign

During the half-day CKD campaign, health education materials (ie, a leaflet as seen in Multimedia Appendix 1, a short textbook as seen in Multimedia Appendix 2, and a recording notebook) were provided to the study patients. A nephrologist facilitated the health campaign: the contents of the textbook and leaflet were discussed, and patients were taught how to measure salt using the spoon during preparation of their daily meals. The research team have diverse professional backgrounds and have established the content of the CKD textbook and leaflet in the patients’ native language (Bangla), based on the educational materials from the website of the National Kidney Foundation, New York, United States, after receiving permission. Important information related to CKD, such as basics of the kidney and kidney diseases, stages of disease, risk factors, and preventive measures, were used to develop the textbook and leaflet in the patients’ native language (Bangla).

#### mHealth Technology

Basic health education information about CKD was included in the content to be delivered through a mobile phone call to help patients gain knowledge and awareness and to improve their behaviors. Discussion about the basics of kidney diseases, risk factors, and preventive measures of CKD was performed by CHWs over a mobile phone call with the study patients. The patients had the liberty to discuss their health-related issues with the CHWs over a period of 10 minutes (Table 2). A nephrologist trained the CHWs in the provision of mHealth education.

**Table 2:** Content of mobile health (mHealth) education that took place over a 10-minute mobile phone call.

Topic	Content of mHealth education
Kidneys	Kidneys are bean shaped and positioned near the middle of your back on either side of your backbone. Your kidneys are part of the body’s urine system. Kidney functions include the following: Remove waste products from the body Remove drugs from the body Balance the body’s fluids Release hormones that regulate blood pressure Produce an active form of vitamin D that promotes strong and healthy bones Control the production of red blood cells
Major risk factors for kidney disease	Some major risk factors include the following: Diabetes High blood pressure Family history of kidney disease, diabetes, or high blood pressure Age 50 years or older Obesity Long-time use of painkillers, such as aspirin and ibuprofen Chronic kidney infection Kidney stones Smoking
Some ways to protect kidneys	Ways to protect kidneys include the following: Keep blood sugar, blood pressure, and cholesterol under control Lose weight, if needed Eat healthy meals Take all medicines as prescribed Get regular exercise Do not smoke Avoid some over-the-counter medications, such as aspirin or ibuprofen, because they can harm kidneys
Diabetes	Diabetes damages your kidneys. Managing blood sugar level slows kidney damage. Some advice includes the following: Maintain a healthy diet Keep a healthy body weight Perform at least 30 minutes of moderate-intensity physical exercise, 5 days per week Take medication regularly if prescribed Monitor your blood sugar regularly
Hypertension	Getting your blood pressure back to normal can reduce kidney damage, and some blood pressure tablets actually protect your kidneys. Some advice includes the following: Reduce salt intake: excess salt in your body causes your blood pressure to go up; this damages your blood vessels and increases the risk of heart attack and stroke. Please consume 5 g of salt per day or less (1 teaspoon) Check blood pressure at regular intervals. If possible, buy a blood pressure monitor and measure your blood pressure at home. This allows you to keep records of your blood pressure and you can see if it changes over time Take medication regularly if prescribed

#### Blood Pressure Check

The CHWs performed home visits where they checked patients’ BP once per week and continued over the study period.

### Control Group

The control group received usual care and were followed up over the study period.

### Sample Size

We assume that the average level of existing knowledge among patients with CKD (stages 1-3) is 40% [24], and that the average level of expected knowledge after the intervention will increase to 70% [25]. Therefore, considering 90% power and 20% loss to follow-up, the total sample size should be 126 (63 in each group). The sample size was estimated based on the following formula:

N = ([p1 × q1 + p2q2] / [p2 – p1]2) × factor for α and β

Here, p1=40%=0.40, the percentage of existing knowledge, and p2=70%=0.70, the percentage of expected knowledge after the intervention. In addition, q1 = 1 – p1 = 0.60; q2 = 1 – p2 = 0.30; power is equal to 90%; and loss to follow-up is equal to 20%.

### Endpoints

The primary outcome is the evaluation of improved scores from the Chronic Kidney Disease Knowledge Questionnaire [24].

The secondary outcomes are as follows:

1. Improved QOL, as measured by the EuroQol 5-Dimension 5-Level (EQ-5D-5L) QOL questionnaire [26].
2. Improvements in the levels of BP, BMI, serum creatinine, FBS, hemoglobin, cholesterol, HDL-c, triglyceride, serum uric acid, BUN, and ACR.

Primary and secondary outcomes were measured at baseline, 3 months, and 6 months for both intervention and control groups, except for the laboratory investigation levels, which were measured at baseline and 6 months.

### Measurements of Knowledge and QOL

#### Knowledge

Knowledge was measured using the Chronic Kidney Disease Knowledge Questionnaire, a 24-item scale with “true,” “false,” and “I don’t know” multiple-choice answer options, designed to assess CKD knowledge in patients with disease at stages 1 to 3. Knowledge scores were calculated by adding the number of correct answers divided by the total number of questions and multiplying by 100 to obtain a percentage score (Multimedia Appendix 3). For all questions, the answer “I don't know” was scored as incorrect.

The translation to Bangla was performed according to the state-of-the-art procedure of forward-backward translation. A physician and a university lecturer, both native speakers of Bangla and fluent in English, translated the questionnaire into Bangla first. The translated Bangla versions were compiled, and a single Bangla forward version was created. This forward version was then translated back into English by a professional translator with experience in medical translation and by one medical doctor who had not been involved in previous steps. The back-translated versions were then compiled and compared by the researcher, and all four versions were submitted to the expert committee that was formed for the validation study. The expert committee developed the questionnaire; pretesting was then conducted at the community level without any knowledge of diagnosis. The suggested changes were made accordingly based on the pretesting responses. Finally, the Bangla version of the CKD knowledge questionnaire was created.

#### Quality of Life

QOL was measured using the standardized EQ-5D-5L questionnaire (Multimedia Appendix 3). The EQ-5D-5L contains five dimensions: mobility, self-care, usual activities, pain or discomfort, and anxiety or depression [26,27]. Each dimension has five levels: no problems, slight problems, moderate problems, severe problems, and unable to or extreme problems. A higher score indicates better QOL [28].

### Ethical Considerations

This study has been approved by the Research Review Board and the Ethical Review Board of icddr,b. The study was registered at ClinicalTrials.gov (NCT04094831). This study is being conducted in accordance with the Declaration of Helsinki [29]. The study objectives; the importance, risks, and benefits of the research; and the patients’ rights were explicitly communicated to all participants before recruitment. Participation was completely voluntary and written informed consent was obtained from all patients. Each study patient’s identity will remain anonymous.

### Statistical Analysis

The intention-to-treat analysis will be performed to compare the outcomes of the intervention and control groups. All baseline indicators at the time of registration will be analyzed to ensure the comparability of the randomized samples. Categorical variables will be expressed as means and SDs and will be analyzed by chi-square tests, discrete variables will be expressed as frequencies and percentages, and continuous variables will be analyzed by *t* tests or Mann-Whitney *U* tests. Multiple comparisons will be performed by two-way analysis of variance tests for the evaluation of the outcome variables, such as CKD knowledge, physical measurements, and QOL at baseline, 3 months, and 6 months. However, outcome variables for laboratory findings were measured at baseline and 6 months. Data will be analyzed using SPSS (version 22.0; IBM Corp) and the significance level will be set at the level of *P*<.05.

## Results

The authors completed patient enrollment in November 2020, and the intervention and data collection were performed from November 2020 to May 2021. The results of the first analysis were made available in July 2021, as expected. We enrolled 126 patients (control group, n=63; intervention group, n=63) in the study. The mean age of the participants was 57.97 (SD 15.03) years for the control group and 57.32 (SD 14.37) years for the intervention group. A total of 71% (45/63) and 60% (38/63) of the patients were female in the control and intervention groups, respectively. A total of 67% (42/63) and 56% (35/63) of the patients were housewives in the control and intervention groups, respectively. A total of 79% (50/63) and 71% (45/63) of patients were married in the control and intervention groups, respectively. Furthermore, regarding the control and intervention groups, 60% (38/63) and 52% (33/63) of the patients were literate, 86% (54/63) and 78% (49/63) of the patients had a monthly income of US $100 per month or higher, 13% (8/63) and 16% (10/63) of the patients were current tobacco users, and 43% (27/63) and 30% (19/63) were current smokeless tobacco users, respectively (Table 3).

**Table 3:** Demographic characteristics among the study participants.

Characteristic		Control group participants (n=63)	Intervention group participants (n=63)
Age in years, mean (SD)		57.97 (15.03)	57.32 (14.37)
**Gender, n (%)**			
	Female	45 (71)	38 (60)
	Male	18 (29)	25 (40)
**Literacy, n (%)**			
	Illiterate	25 (40)	30 (48)
	Literate	38 (60)	33 (52)
**Occupation, n (%)**			
	Housewife	42 (67)	35 (56)
	Farmer	3 (5)	4 (6)
**Marital status, n (%)**			
	Married	50 (79)	45 (71)
	Widowed	12 (19)	18 (29)
**Income (US $), n (%)**			
	<100/month	9 (14)	14 (22)
	≥100/month	54 (86)	49 (78)
**Current tobacco smoker, n (%)**			
	Yes	8 (13)	10 (16)
	No	55 (87)	53 (84)
**Current smokeless tobacco user, n (%)**			
	Yes	27 (43)	19 (30)
	No	36 (57)	44 (70)

## Discussion

### Overview

This study’s overall effectiveness will be enhanced by the publication of this research protocol. A nephrologist-facilitated health campaign aims to increase knowledge and raise awareness about CKD-related health concerns by engaging people in discussions about important and influential health information. The aim of such events is to increase knowledge and understanding about CKD and its related risk factors, as well as to educate people on how they can avoid disease progression by living a healthy lifestyle. In addition, mHealth has been shown to enhance awareness, QOL, and behavior among low-income patients with chronic diseases, such as diabetes, and is currently being tested among low-income CKD patients [30]. While CKD management is a significant burden on health practitioners in low-capital settings, CHWs can be an important and underutilized resource for patient education. CHWs have been shown to be effective in assisting people in improving their health habits. A CHW intervention among patients with diabetes, for example, increased patients’ awareness and helped them control their blood glucose levels and BP [31]. To the best of our knowledge, this is the first research study in Bangladesh to evaluate the outcome of health education through a CKD campaign and mobile phone calls to reduce CKD-related burden. mHealth shows a lot of potential in terms of raising patient awareness and understanding of kidney disease, as well as improving kidney knowledge [16]. Furthermore, studies from both developed and developing countries have demonstrated the efficacy and user acceptance of mHealth technologies in the management of CKD among patients; however, the majority of them were limited to dialysis patients [14].

### Strengths

A nephrologist facilitated the health campaign in this study, and CHWs provided health education through mobile phone calls in the patients’ native language (Bangla). Health education materials were developed using the same language for better understanding, even with minimum technical knowledge and skills. Furthermore, the study’s strengths include the unbiased systematic sampling approach used to recruit patients and the standard laboratory facility used to identify patients with CKD.

### Limitations

The patients in our study were randomly selected from the three unions of the Mirzapur subdistrict, and this does not represent the entire rural and periurban CKD population. In addition, data contamination from study patients and family members could be another study limitation. Therefore, the CHWs received verbal consent from patients not to disclose any study details to their neighbors. Finally, due to budgetary limitations and time constraints, our 6-month follow-up duration was relatively brief.

### Conclusions

If our results show an enhancement of the study outcomes among patients with CKD (stages 1-3), we suggest integrating health education via a campaign and mHealth as effective tools at the national level. Further, we can improve patient knowledge and motivate patients with CKD regarding their health practices to improve their QOL.

## Appendix

Multimedia Appendix 1 Health education material: leaflet.

Multimedia Appendix 2 Health education material: short textbook.

Multimedia Appendix 3 Questionnaires.

## Abbreviations

ACR: albumin to creatinine ratio
BP: blood pressure
BUN: blood urea nitrogen
CHW: community health worker
CKD: chronic kidney disease
CONSORT: Consolidated Standards of Reporting Trials
DSS: demographic surveillance system
EQ-5D-5L: EuroQol 5-Dimension 5-Level
ESRD: end-stage renal disease
FBS: fasting blood sugar
HDL-c: high-density lipoprotein cholesterol
LMIC: low- and middle-income countries
mHealth: mobile health
PHC: Portable Health Clinic
QOL: quality of life
SPIRIT: Standard Protocol Items: Recommendations for Interventional Trials
