# Supplementary figures and images for: Health Education Through a Campaign and mHealth to Enhance Knowledge and Quality of Life Among Patients With Chronic Kidney Disease in Bangladesh: Protocol for a Randomized Controlled Trial
Source: JMIR Res Protoc. 2021 Nov 19;10(11):e30191. doi: 10.2196/30191 (PMC8663577; doi:10.2196/30191)

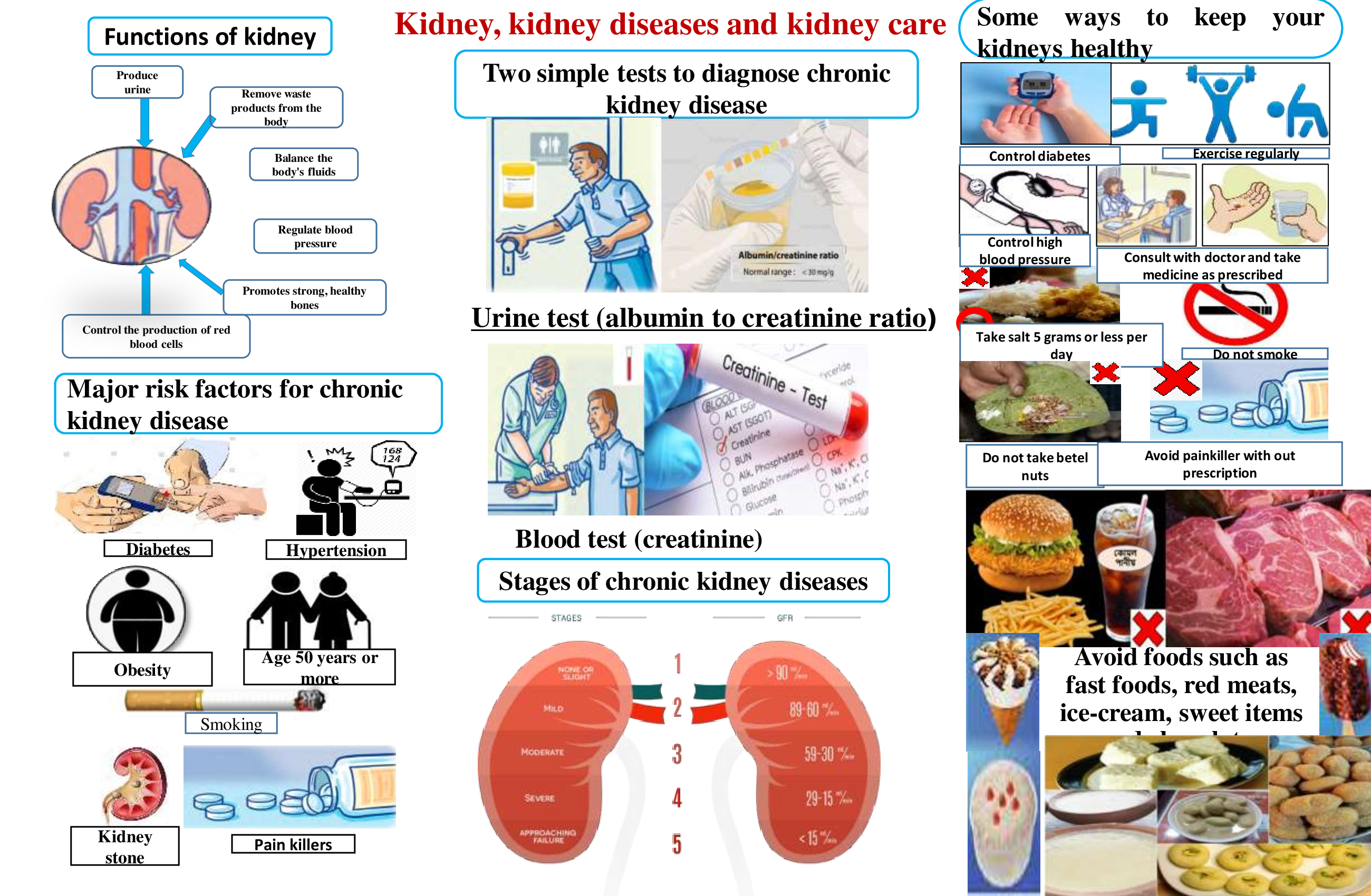

Supplement: Multimedia Appendix 1 [file resprot_v10i11e30191_app1.png]
