# Supplementary material for: Health Education Through a Campaign and mHealth to Enhance Knowledge and Quality of Life Among Patients With Chronic Kidney Disease in Bangladesh: Protocol for a Randomized Controlled Trial
Source: JMIR Res Protoc. 2021 Nov 19;10(11):e30191. doi: 10.2196/30191 (PMC8663577; doi:10.2196/30191)
Supplement: Multimedia Appendix 2 [file resprot_v10i11e30191_app2.pdf]

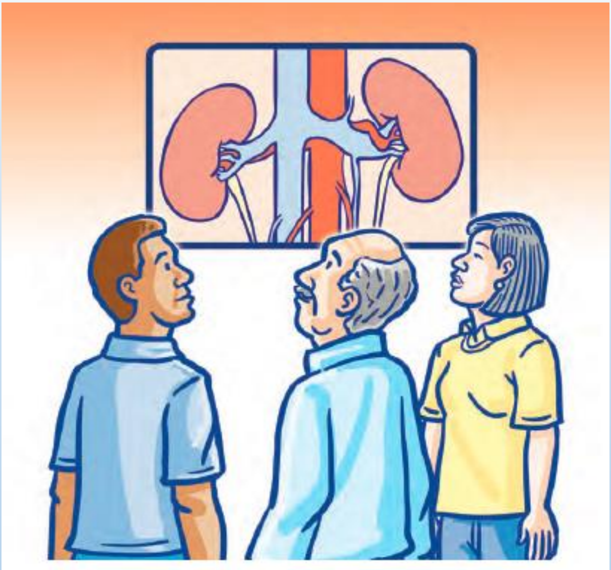

Kidney, kidney disease and kidney care

Protocol Title- Health education through a campaign and mHealth to enhance knowledge and improve quality of life among patients with Chronic Kidney Diseases in rural and peri-urban Bangladesh

What is Kidney?

Kidneys are bean shaped and positioned near the middle of your back on either side of your backbone. Your kidneys are part of the body’s urine system.

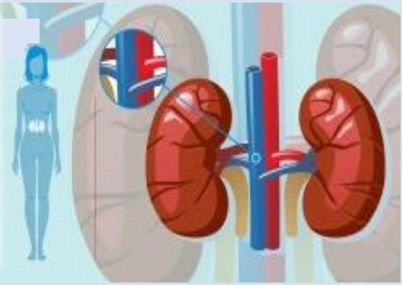

Why are the kidneys so important?

- Most people know that a major function of the kidneys is to remove waste products and excess fluid from the body. These waste products and excess fluid are removed through the urine.

- The critical regulation of the body's salt, potassium and acid content is performed by the kidneys. The kidneys also produce hormones that affect the function of other organs. For example, a hormone produced by the kidneys stimulates red blood cell production. Other hormones produced by the kidneys help regulate blood pressure and control calcium metabolism.

Functions of Kidney

- Remove waste products from the body
- Remove drugs from the body
- Balance the body's fluids
- Release hormones that regulate blood pressure
- Produce an active form of vitamin D that promotes strong, healthy bones
- Control the production of red blood cells

What is chronic kidney disease?

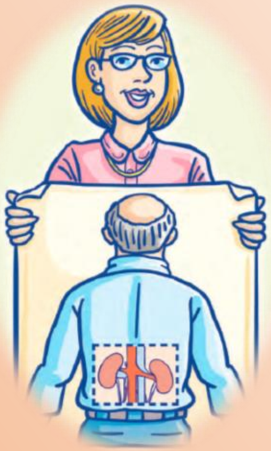

Chronic kidney disease includes conditions that damage your kidneys and decrease their ability to keep you healthy by doing the jobs listed. If kidney disease gets worse, wastes can build to high levels in your blood and make you feel sick. You may develop complications like high blood pressure, anemia (low blood count), weak bones, poor nutritional health and nerve damage. Also, kidney disease increases your risk of having heart and blood vessel disease. These problems may happen slowly over a long period of time. Chronic kidney disease may be caused by diabetes, high blood pressure and other disorders. Early detection and treatment can often keep chronic kidney disease from getting worse. When kidney disease progresses, it may eventually lead to kidney failure, which requires dialysis or a kidney transplant to maintain life.

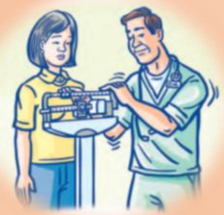

Causes of chronic kidney diseases

The two main causes of chronic kidney disease are diabetes and high blood pressure, which are responsible for up to two-thirds of the cases. Diabetes happens when your blood sugar is too high, causing damage to many organs in your body, including the kidneys and heart, as well as blood vessels, nerves and eyes. High blood pressure, or hypertension, occurs when the pressure of your blood against the walls of your blood vessels increases. If uncontrolled, or poorly controlled, high blood pressure can be a leading cause of heart attacks, strokes and chronic kidney disease. Also, chronic kidney disease can cause high blood pressure.

Other conditions that affect the kidneys are:

- Kidney infection
- History of Kidney disease
- Kidney stone or tumor
- Painkiller

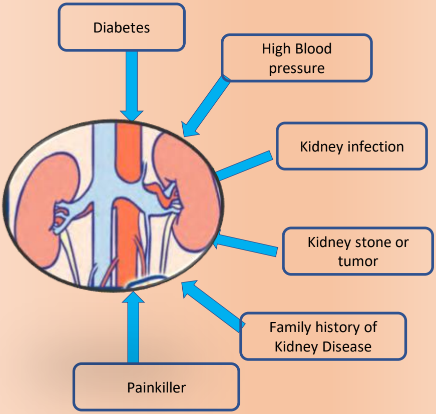

What are the symptoms of CKD?

Most people may not have any severe symptoms until their kidney disease is advanced. However, you may notice that you:

- feel more tired and have less energy
- have trouble concentrating
- have a poor appetite
- have trouble sleeping
- have muscle cramping at night
- have swollen feet and ankles
- have puffiness around your eyes, especially in the morning
- have dry, itchy skin
- need to urinate more often, especially at night.

Major risk factors for chronic kidney disease

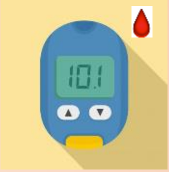

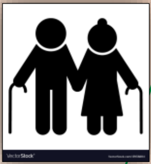

- Diabetes
- High blood pressure
- Family history of kidney disease, diabetes or high blood pressure
- Age 50 years or above
- Obesity
- Low birth weight
- Long time use of painkillers such as Aspirin, Ibuprofen etc.
- Chronic kidney infection
- Kidney stone
- Smoking

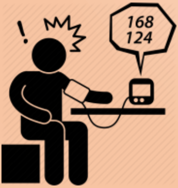

Kidney disease can be treated

- Kidney disease does not happen overnight. It happens slowly over many years, and in stages.
- There are five stages of kidney disease. In each stage, the kidneys don't work as well as the stage before.
- With treatment and lifestyle changes, you can help slow or stop your kidney disease from getting worse.

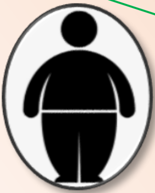

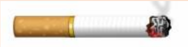

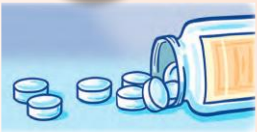

Stages of chronic kidney disease

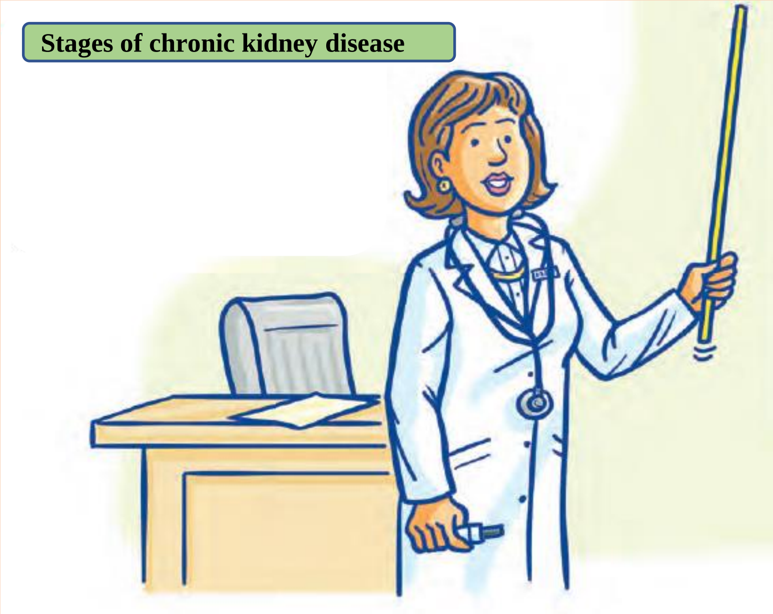

Stages of chronic kidney disease		GFR*	% of kidney function
Stage 1	Kidney damage with normal kidney function	90 or higher	90-100%
Stage 2	Kidney damage with mild loss of kidney function	89 to 60	89-60%
Stage 3a	Mild to moderate loss of kidney function	59 to 45	59-45%
Stage 3b	Moderate to severe loss of kidney function	44 to 30	44-30%
Stage 4	Severe loss of kidney function	29 to 15	29-15%
Stage 5	Kidney failure	Less than 15	Less than 15%

*** Your GFR number tells you how much kidney function you have. As kidney disease gets worse, the GFR number goes down.**

Two simple tests to check chronic kidney disease

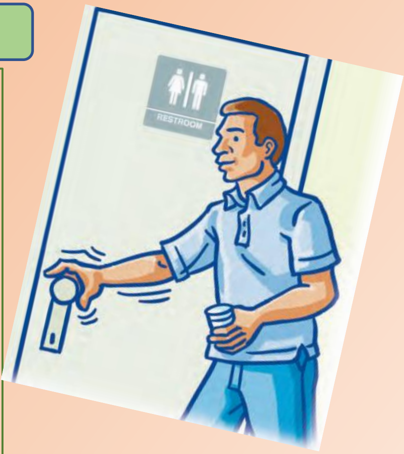

Blood Test: Glomerular Filtration Rate (GFR)

The GFR tells how well your kidneys are working to remove wastes from your blood. It is the best way to check kidney function. Over 90 is good, 60-89 should be monitored, less than 60 for 3 months indicates kidney disease.

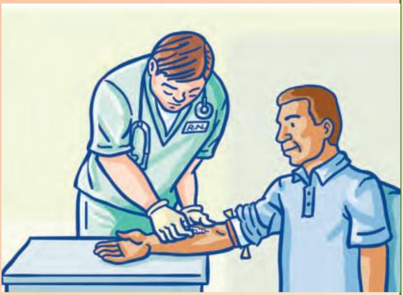

Urine Test: Albumin Creatinine Ratio (ACR)

You could help save your kidneys with a simple urine test. Ask your doctor about Albumin Creatinine Ratio (ACR) which estimates the amount of a type of protein, albumin, that is in your urine.

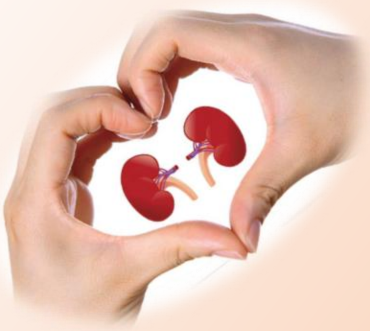

Some ways to protect kidneys

- Keep blood sugar, blood pressure, and cholesterol under control
- Lose weight, if needed
- Eat healthy meals
- Take all medicines as prescribed
- Get regular exercise
- Don’t smoke
- Limit alcohol
- Avoid some over-the-counter medicines (such as aspirin, or ibuprofen) because they can harm kidneys

Diabetes

Diabetes is the main risk factor for chronic kidney disease. Diabetes damages your kidney. Managing blood sugar level slows the kidney damage.

Advise

- Take a healthy diet
- Keep a healthy body weight
- Exercise at least 30 minutes of moderate physical exercise on 5 days a week
- Take medicine regularly if prescribed
- Monitor your blood sugar regularly

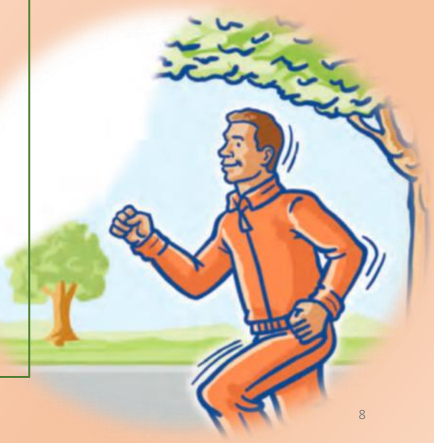

High Blood pressure

Getting your blood pressure back to normal can reduce your kidney damage and some blood pressure tablets actually protect your kidney.

Advise:

Reduce salt intake: Excess salt in your body causes your blood pressure to go up. This damages all your blood vessels and increases the risk of heart attack and stroke. Please take salt less than 5gm/day (1 tea spoon)

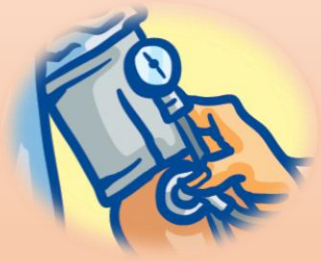

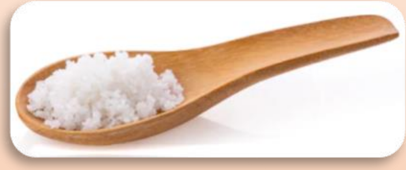

- Consult with your doctor and take medicine regularly if prescribed

- Check blood pressure in regular interval. It is possible then buy a blood pressure monitor and measure your blood pressure at home. This can allow you to keep records of your blood pressure and you can see if your blood pressure changes over time

How Is Kidney Disease Treated?

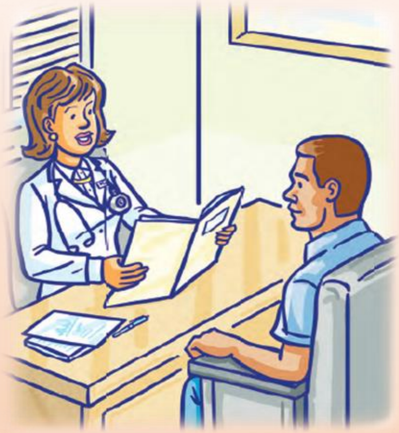

Chronic kidney disease can't be cured, but it can be treated:

- Chronic kidney disease needs to be treated
- The earlier kidney disease is found, the earlier it can be treated
- The treatment plan depends on the stage of kidney disease and other health problems
- The goals of treatment are to slow kidney disease and keep it from getting worse

Did you know?

More people with kidney disease die of heart disease before their kidneys fail? This is because kidney disease causes heart and blood vessel problems. People with or without kidney disease can help lower their risk of heart and blood vessel. So, make heart-healthy and kidney-healthy choices:

• Don't smoke	• Eat healthy meals
• Control high blood pressure	• Exercise regularly
• Control diabetes	• Lose weight if you are overweight
• Control cholesterol.	• Follow your doctor's instructions and take medicines as prescribed

What Could Happen if Kidney Disease is Not Treated?

If kidney disease is not treated, you have a higher risk of:

- High blood pressure (if you didn't have it before)
- Heart and blood vessel disease
- Anemia
- Mineral and bone disorder
- Kidney failure and the need for kidney transplant or dialysis
- Other health problems

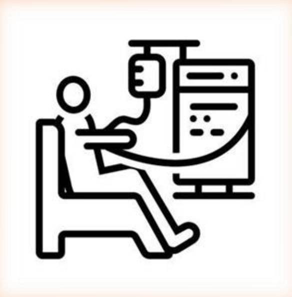

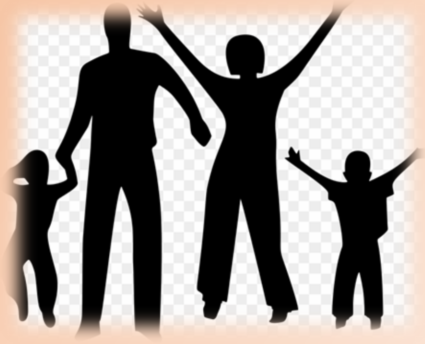

Keep your kidneys healthy

Acknowledgement

NATIONAL KIDNEY FOUNDATION.
