# Supplementary material for: Health Education Through a Campaign and mHealth to Enhance Knowledge and Quality of Life Among Patients With Chronic Kidney Disease in Bangladesh: Protocol for a Randomized Controlled Trial
Source: JMIR Res Protoc. 2021 Nov 19;10(11):e30191. doi: 10.2196/30191 (PMC8663577; doi:10.2196/30191)
Supplement: Multimedia Appendix 3 [file resprot_v10i11e30191_app3.pdf]

Title: Health education through a campaign and mHealth to enhance knowledge and improve quality of life among patients with chronic kidney diseases in rural and peri-urban Bangladesh.

Questionnaire:

A. Identification (Age, gender, marital status, occupation, educational background, income/month, wealth quantile)

1. Questionnaire ID number<br>[Start with questionnaire number as '001']				7. Researcher/Interviewer ID number<br>[Use your personal ID]		
2. Interview date				8. Interview Duration		
	Day	Month	Year		Start	End	Duration (min)
3. Union (name & code)		Code		9. Staff Code		
				10. Status of Interview		1=Complete 2=Incomplete
4. Village name				**If incomplete, write the reason:**		
5. Respondent Name						
6. Contact no./Mob no.						
11. Date of birth [In years]	Day	Month	Year			
12. Gender		[**Code:** 1=Male, 2=Female]				
13. Education	What is the highest grade of your education?		[**Code:** 1=Cannot read & write, 2= Can sign only, 3= Can read only, 4= Can read & write, 5=Class I-V, 6= Class VI-X, 7= SSC, 8= HSC, 9= Bachelor, 10= master's & above, 33=Religious school]			
15. Marital Status	What is your marital status?		[**Code:** 1=Single, 2=Married, 3=Widow, 4=Never married, 5=Separated, 6=Divorced, 7=Other (specify): ________________, 77=Don't know, 88=Refused]			

B. Respondent's Household [HH] information (socio-economic variables)

16. HH Size	How many people live in your HH?		[Number only<br>**Interviewer note** – This means living there continuously for the **past 6 months**]
17. Occupation	What is your primary occupation?		[**Code:** 1=Farmer, 2=Day laborer, 3=Share cropper, 4=Rickshaw/push cart puller, 5=Taxi/bus/truck/tempo driver, 6=Mill/industry worker, 7= Skilled worker, 8=Office non-

			executive, 9=Office executive, 10=Petty business, 11=Big business, 12=Overseas employment, 13=Boatman, 14=Fisherman, 15=Unemployed, 16=Beggar, 17=Street vendor, 18=Housewife, 19=Retired,<br>20=Other (specify): ___________________,<br>77=Don't know, 88=Refused]
18.1 Income	Who is the income earner of your HH?		[**Code:** 1= Only HH head, 2= HH head and others, 3=Others] Mention your relationship with him/her ______________________________
18.2 Income	What are the main sources of your HH income?		[**Code:** 1= Unemployed, 2= Unpaid household work, 3= Government service, 4= Private service, 5= Agriculture labor, 6= Non-agriculture labor, 7= Agriculture production, 8=Trading agent, 9= House tutoring, 10= Remittance, 11= Pension, 12= Rental, specify if others]
18.3 Income	How much money does your household earn in every month [average in BDT]?		Tk. per month
18.4 Expense	How much money does your household expense in every month [average in BDT]?		Tk. per month

C. Current medical History, medication and adherence

19.	Have you ever been diagnosed with CKD?		[**Code:** 1=Yes, 2= No, 77=Don't know, 88=Refused]
20.	Do you suffer any other diseases, such as hypertension, Diabetes, stroke, heart disease, obesity etc.?		[**Code:** 1=Yes, 2= No, 77=Don't know, 88=Refused]<br><br>If yes, then what was diagnosed-----------------------<br>Mention the relationship with you_____________
21.	Do you take all the prescribed medications for it?		[**Code:** 1=Yes, 2=No]
22.	How many different medicines you are taking in a day?		[***Interviewer note:*** Mention *approx.* number]
23.	Do you take medicine regularly?		[**Code:** 1=Yes, 2=No]
24.	Why you do not take them as prescribed?		[**Code:** 1=I cannot afford the cost, 2=Medications are not easily available, 3=I do not like to take medications, 4=I only take them

			when I feel that I need them, 5=I do not like the side effects of the medication, 6= I prefer alternative medicine, 7=I forget, 8= Other______________________, 77=Don't know]
25.	Are you having any problems taking your medications?		[**Code:** 1=Yes, 2=No], Specify if 'Yes'___________________________
26.	Do you take any herbal medicine?		[**Code:** 1=Yes, 2=No], Specify if 'Yes'___________________________

D. History of CKD

27.	How did you come to know about your CKD?		[**Code:** 1=In a routine medical control, 2=Screening program, 3=Emergency service, 4=Other (specify: _______________), 77=Don't know, 88=Refused]
28.	At what age were you diagnose as CKD?		[**Code:** Mention it in year / age: ______________, 77=Don't know, 88=Refused]
29.	Where did you confirm your first diagnosis of having CKD?		[**Code:** 1=This primary health center, 2=Other primary care clinic/physician, 3=Secondary care hospital*, 4=Tertiary care hospital*, 5=At a pharmacy/drugstore, 6=Other (specify_________), 77=Don't know, 88=Refused]<br>* manual explains what it is meant

E. Behavioural questionnaire:

30.	Do you smoke cigarettes?		[**Code:** 1=Yes, 2=No] If 'yes'
31.	How many cigarettes do you smoke a day?		[**Code:** 77=Don't know, 88=Refused] [***Interviewer note:*** Mention *approx.* number]
32.	How many years have you been smoking?		[**Code:** 77=Don't know, 88=Refused] [***Interviewer note:*** Mention years only]
33.	Does anyone smoke in your house?		[**Code:** 1=Yes, 2=No, 77=Don't know, 88=Refused]
34.	Did you ever take smokeless tobacco? (Zarda, Paan, tobacco leaf, Gul, Sadapata, Paan Masala etc)		[**Code:** 1=Yes (go to next question), 2=No, 77=Don't know, 88=Refused] [***Interviewer note:*** *Such as-* Zarda, Paan with tobacco leaf, Gul, Sadapata, Pan masala, Nasshi]
35.	How often do you take smokeless tobacco?		[**Code:** 1=Every day, 2=At least once a week, 3=At least once in the last month, 4=Not at all, 77=Don't know, 88=Refused] [***Interviewer note:*** Data of last 30 days]
36.	How many times you		[***Interviewer note:*** Mention *approx.*

	take it a day?		number]
37.	Do you take alcohol?	Code:	[**Code:** 1=Yes, 2=No] if yes, go to the next Q.
38.	How often do you take alcohol?	Code:	1= Daily, 2= 5-6 days per week, 3= 1-4 days per week, 4= 1-3 days per month, 5= Less than once a month
39.	How much do you take alcohol in a day (standard drink)?		Mention it in ml___________
40.	What type of physical activity you are currently involved?		[Code: 1=Bicycling, 2=Lifting Heavy loads, 3=Swimming, 4=Walking, 5= Digging, 6= Construction work, 7= Harvesting, 8=Others ___________________, 10=None]
41.	How often do you do physical activity?		[Code: 1=1-3 times a week, 2=3-5 times a week, 3=5-7 times a week, 4=Inconsistently, 5=None]
42.	How many hours do you sleep in a day?		Mention it in hours_______
43.	Do you have any sleeping disturbance?		[Code: 1=Yes (go to next question), 2=No, 77=Don't know, 88=Refused]

F. Family History

44.	Have any of your family members ever been diagnosed with CKD?		[**Code:** 1=Yes, 2= No, 77=Don't know, 88=Refused]
45.	Does your family have a history of diagnosed such as hypertension, Diabetes, obesity etc.? If yes, who was diagnosed?		[**Code:** 1=Yes, 2= No, 77=Don't know, 88=Refused]<br><br>If yes, then what was diagnosed-----------------------<br>Mention the relationship with you_____________
46.	Have any of your family members ever been hospitalized due to complication of CKD related complication?		[**Code:** 1=Yes, 2= No, 77=Don't know, 88=Refused]

G. Clinical Measures

Height				**Cm**	Blood pressure							
Weight				**Kg**	Measurement 1 (Right arm)							mmHg
Waist circumference				**Cm**	Measurement 2 (Right arm)							mmHg
Hip circumference				**Cm**	Measurement 1 (Left arm)							mmHg
Pulse				**Per/min**	Measurement 2 (Left arm)							mmHg

H. Medical Record and Recording sheet:

I would like to do some investigations to see some of your results for Chronic kidney diseases. This will help us to understand the diagnosis and risk factors of CKD. I would like to remind you that this will be strictly confidential.

**Test**	**Value**	**Test**	**Value**	**Date of test (DD/MM/YYYY)**
**Blood**				
Fasting blood sugar (FBS)	mmol/L	S.Creatinine	mg/dl	
S.Cholesterol	mg/dl	HDL-c	mg/dl	
S.Triglyceride	mg/dl	S.Albumin	gm/dl	
Hb	g/dl			
Blood urea nitrogen (BUN)		S.Uric acid		
**Urine sample**				
ACR	mg/gm			
**Salinity**				
Food salinity				
Urinary salinity				

I. Chronic Kidney Disease Knowledge Questionnaire

For Sections, 1-5, please answer ‘True’, ‘False’ or ‘I don’t know’ to the following questions:

Section 1

No	Question	True	False	I don’t know
1	A person can lead a normal life with one healthy kidney.	☐	☐	☐
2	Herbal supplements can be effective in treating chronic kidney disease.	☐	☐	☐
3	Certain medications can help to slow-down the worsening of chronic kidney disease.	☐	☐	☐

Section 2 What functions do the kidney perform in our body?

No	Question	True	False	I don’t know
4	The kidneys make urine.	☐	☐	☐
5	The kidneys clean blood.	☐	☐	☐
6	The kidneys help to keep blood sugar level normal.	☐	☐	☐
7	The kidneys help to maintain blood pressure.	☐	☐	☐
8	The kidneys help to breakdown protein in the body.	☐	☐	☐
9	The kidneys help to keep the bones healthy.	☐	☐	☐

Section 3 Which of the following are commonly used to determine the health of your kidneys?

No	Question	True	False	I don’t know
10	A blood test.	☐	☐	☐
11	A urine test.	☐	☐	☐
12	A faecal (poo) test.	☐	☐	☐
13	Blood pressure monitoring.	☐	☐	☐

Section 4 What are the risk factors for chronic kidney disease?

No	Question	True	False	I don’t know
14	Diabetes.	☐	☐	☐
15	Being female.	☐	☐	☐
16	High blood pressure.	☐	☐	☐
17	Heart problems such as heart failure or heart attack.	☐	☐	☐
18	Excess stress.	☐	☐	☐
19	Obesity.	☐	☐	☐

Section 5 What are the signs and symptoms that a person might have if they have advanced chronic kidney disease or kidney failure?

No	Question	True	False	I don’t know
20	Water retention (excess water in the body) (e.g. edema in leg)	☐	☐	☐
21	Fever.	☐	☐	☐
22	Nausea/vomiting.	☐	☐	☐
23	Loss of appetite.	☐	☐	☐
24	Increased fatigue (tiredness).	☐	☐	☐

J. **Quality of Life questionnaire-**

1	MOBILITY		1=I have no problems in walking about ☐<br>2= I have slight problems in walking about ☐<br>3= I have moderate problems in walking about ☐<br>4= I have severe problems in walking about ☐<br>5= I am unable to walk about ☐
2	SELF-CARE		1=have no problems washing or dressing myself ☐<br>2= I have slight problems washing or dressing myself ☐<br>3= I have moderate problems washing or dressing myself ☐<br>4= I have severe problems washing or dressing myself ☐<br>5= I am unable to wash or dress myself ☐
3	USUAL ACTIVITIES (e.g. work, study, housework, family or leisure activities)		1=I have no problems doing my usual activities ☐<br>2= I have slight problems doing my usual activities ☐<br>3= I have moderate problems doing my usual activities ☐<br>4= I have severe problems doing my usual activities ☐<br>5= I am unable to do my usual activities ☐
4	PAIN / DISCOMFORT		1=I have no pain or discomfort ☐<br>2= I have slight pain or discomfort ☐<br>3= I have moderate pain or discomfort ☐<br>4= I have severe pain or discomfort ☐<br>5= I have extreme pain or discomfort ☐
5	ANXIETY / DEPRESSION		1=I am not anxious or depressed ☐<br>2= I am slightly anxious or depressed ☐<br>3= I am moderately anxious or depressed ☐<br>4= I am severely anxious or depressed ☐<br>5= I am extremely anxious or depressed ☐
NB: There should be only ONE response for each dimension<br>NB: Missing values can be coded as ‘9’.<br>NB: Ambiguous values (e.g. 2 boxes are ticked for a single dimension) should be treated as missing values.			
